# Exploring the changing of name as a socio-cultural adaptation strategy of the Javanese Diaspora in Sabah Borneo—Malaysia

**DOI:** 10.3389/fsoc.2025.1487934

**Published:** 2025-02-07

**Authors:** Sahid Teguh Widodo, Suyatno Suyatno, Bahtiar Mohamad, Shafinar Ismail

**Affiliations:** ^1^Faculty of Cultural Sciences, Universitas Sebelas Maret, Surakarta, Central Java, Indonesia; ^2^Othman Yeop Abdullah Graduate School of Business (OYAGSB), Universiti Utara Malaysia, Kuala Lumpur, Malaysia; ^3^Faculty of Business and Management, Universiti Teknologi MARA, Melaka, Malaysia

**Keywords:** personal name, diaspora, Java, Sabah, Malaysia

## Abstract

This research explores the evolution of personal names within the Diaspora Jawa Sabah (DJS) community, focusing on how historical events, social changes, and cultural practices have influenced DJS names, particularly after Sabah joined Malaysia in 1963. Using content analysis and in-depth interviews, the study investigates the migration history and cultural factors shaping the linguistic aspects of DJS names in Sabah. The findings reveal four key points: (1) the distinct migration histories of Javanese people to Sabah compared to the Malaysian Peninsula, (2) the impact of historical, social, and cultural phenomena on DJS names before and after 1963, (3) the transformation of DJS names into modern Malay Islamic names reflecting personal aspirations, and (4) the collective cultural and ideological shifts within the DJS community. This research contributes to the understanding of personal names as a reflection of cultural identity, illustrating the dynamic relationship between linguistic forms and broader social, historical, and cultural contexts. The study enhances the theoretical and practical knowledge of naming practices as indicators of societal changes within the DJS community.

## 1 Introduction

Akinnaso (1980), in his writing entitled “The Sociolinguistic Basis of Yoruba Personal Names” states that personal names are related to issues outside the linguistic aspect, for example, related to conceptions of time, descriptions of places, atmosphere, or events, social status, history, and certain traditions that have been distilled in unique ways. The language form of proper names has a distinctive sound system and, therefore, can be used by a person or social group to get to know each other, communicate, and work together. This statement is in line with Xu (2020) opinion that self-names are produced by society and are also a certain “social code” that can explain various things about society itself. This means that self-names are not only related to sensory elements but involve enthusiasm, wishes, desires, and character, which refer to abstract ideas of society's culture (Cavallaro, 2001).

Until now, people often understand proper names only as a form of aesthetic language given to someone in the form of special words, terms, or expressions that can be used as someone's identity or something from another (Hofmann, 1993; Widodo et al., 2023). So, it is not surprising that a proper name is recognized as an identity that differentiates a person from other people, animals, places, and other things that are known or discussed (Langendonck, 1985). This phenomenon applies to almost all cultures throughout the world (Thomas and Samjose, 2022).

There is still very little public attention from researchers and academics in Indonesia regarding personal names. On the other hand, mono- and multi-disciplinary linguistic research and studies are very much carried out. In fact, it has reached a fairly ideal level of achievement. Uhlenbeck (1982) states several possible reasons, one of which is that research on Javanese proper names is less interesting, narrow, and dry because there is not much that can be explored and revealed. In fact, studies of people's names throughout the world still place the subject of proper names into a single paradigm of linguistic structure (descriptive linguistics). As a result, name research falls into narrow and dry terrain because it does not provide options for other points of view (Crystal, 1987) and is tautological (Moore, 1954). However, in language logic, the two have different basic meanings (Charlesworth, 1959).

Giving one's name is an important part of Javanese culture. The tradition of giving names has lived and developed for centuries, so Javanese culture has very interesting and complete conventions and traditions. This is what triggers the impression that Javanese culture is an ancient culture that is known by the world community as a unique, rich, and beautiful culture (*swarna dwipa*). Expanses of very fertile tropical land, rice barns, and various types of spices grow with the earth's abundant mineral resources and content, giving birth to great traditions and cultural works. Large kingdoms such as Majapahit, Mataram, Pajang, and Demak Bintoro were established in Central Java and became cultural centers in Southeast Asia and Asia in general (Rodhiyah and Hidayat, 2019). One of the great legacies of Javanese culture is the well-organized naming system. A proper name gives birth to new types of names, including title names, nicknames, *paraban* names, pseudonyms, etc. What is interesting is that in Java, various types of people's names are always written in capital letters as a form of respect for the bearer (Widodo et al., 2023).

The linguistic and socio-cultural phenomena of the names of people of Javanese descent in Sabah are no less interesting than those in the Malaysian Peninsula. Historical sources state that since the early 18th century AD in the Sabah region of Borneo, small groups of Javanese people have been found (Abdullah, 2020). It's just that it hasn't appeared much on the surface, most likely because the presence of Javanese people in Sabah, Borneo has spread throughout the country from the Tanjung Datu area to the Lawas area, Sabah. This situation is also different from that in Sarawak and Kuching, where Javanese descendants live in certain areas such as Kampong Haji Baki, Kampong Sekedup, and Kampong Matang, which are often referred to as the collective Kolong-Kolong (Ahmed and De Silva, 2021; Zainuddin, 2024).

The second source stated that initially, the existence of Javanese people in North Borneo and Sabah received little attention from researchers because their mainstream views were still focused on the rise of the Sabah Malaysians who came from Sumatra, Kalimantan, and Bugis-Makassar. These three ethnicities are considered to have played a major role in the growth of Sabah Malay history. The conversation changed when, from various research and reviews, it was felt that Javanese participation was quite strong and always present at every event. In 1880–1930 AD the arrival of Javanese people in Sabah became greater and brought increasingly clear social influence. They came as workers on rubber, oil palm, and pepper plantations (Allport, 1937) or as factory workers during the reign of Charles Brooke until they were passed down to his son C. Vyner Brooke (Sariyan, 2015). It is not surprising that Alatas (2013) states that Malaysia is a “big home” for Javanese travelers and workers outside Indonesia.

The Javanese Peranakan community in Sabah has gradually been assimilated with other ethnic groups, such as the Kadazan-Dusun, Murut, Bajau, Malay, and Chinese (Amat, 2021). The language they use over time also changes. No longer using Javanese, but following ethnic languages and Malaysian language in general (Hitam, 1970). This is the reason why, after living and settling in the territory of the state of Sabah for more than 10 years, Javanese descendants finally received recognition from the Malaysian Kingdom of Sabah; they were no longer considered any descendants but had become a new designation as ethnic Javanese Malays under the law. Malaysian law (Yahya et al., 2017). Some preliminary data shows that a group of Islamic religious teachers from Java have obtained their rights as Malaysian citizens in Sabah and have received the right to occupy land in the Sabah region with Building Use Rights status.

Research related to changing the form of names as a socio-cultural adaptation strategy for people of Javanese descent in Sabah, Borneo, Malaysia, is a prominent constructive effort. Sabah was chosen as a post-research location in Peninsular Malaysia—covering the areas of Johor, Selangor, and Melaka—(Widodo et al., 2023), which is certainly very interesting because of the clearly different backgrounds between the two. Of course, this is also a “leap” from the research report of Suranto (1983) entitled “Study of Javanese names” and Suharno (1987) entitled “Proper names in Javanese society”. Apart from that, it is also a development of the research concept of Shanmuganathan (2021) and Akinnaso (1980). Both emphasize their studies on cultural differences from a cross-cultural perspective (Widodo et al., 2023). This research considers secondary data from De Silva and Edinin (2021)'s article and several additions from Thomas and Samjose (2022), Waldispühl and Wallis (2021), and Khoa (2022).

Another consideration that is quite interesting is the difference in the causes of the movement of Javanese people to Sabah, namely the influence of history, the influence of sending workers by British companies to Sabah, the influence of companies during the Dutch Colonial period, and also the influence of “*romusha*”, namely the system forced labor by Japanese colonialists (1942–1945) in Java. These four causes are used as a fulcrum in uncovering influences outside the linguistic area. Anyway, to this day, these factors still have a psychological influence on the lives of Javanese Peranakans in Sabah (Widodo, 2010). Once again, this is an interesting reason why the self-names of the Malaysian Javanese diaspora need to be researched in depth because they are related to life, thoughts, ideals and individual and collective desires. Apart from that, this research has an important meaning for the development of multi-disciplinary research in the field of linguistics, in addition to understanding the development of cultural tastes, traditions, and worldviews of the Javanese diaspora community in Sabah, Malaysia.

The study critically examines the influence of the historical migration of Javanese people to Sabah on the variations in name forms that emerged; it details the significant impact of historical, social, and cultural phenomena on DJS (Javanese Diaspora in Sabah) names before and after Sabah's integration into Malaysia (1963). It illustrates the evolutionary process of DJS names transforming into modern and contemporary Malay-Islamic names that reflect personal aspirations. The study provides new insights into onomastic studies regarding the influence of cultural and ideological shifts within the DJS community in Sabah, Malaysia. Clearly, despite differences in place, time, and events, this research correlates with theories and concepts established in previous studies (Akinnaso, 1980; Xu, 2020; Cavallaro, 2001; Widodo et al., 2023).

### 1.1 Theoretical basis

#### 1.1.1 People of Javanese descent in Sabah

The migration of Javanese people to Sabah has actually been an issue for centuries. Researchers also put forward many reasons regarding the causes of migration, including the wandering traditions of maritime communities, economic motivations, history, politics, labor issues, marriage, the spread of Islam, and many others. This problem has attracted the attention of experts since the beginning, including research by Werren (1985), Sintang (2005), Tamring and Mahali (2020), and Mugiman et al. (2020). They concluded that the migration issue was caused by (1) the very rapid economic development in Sabah since the BNBC era, (2) the issue of labor requirements in plantations, (3) the issue of Sabah's internal state politics and state policy on the Peninsula, (4) the issue of safety, and several other completely different causes (Dollah and Abdullah, 2017).

The history of the movement of Javanese people has been widely written in every destination country, including Suriname, New Caledonia, South Africa, Sri Lanka, Thailand, Peninsular Malaysia and Borneo, Vietnam, the Philippines, etc. The era between 1880 and 1920 was an important time period for the movement of Javanese people to “new regions” outside Java, namely Sabah-Borneo. There were at least three colonial periods that had an influence on the migration of Javanese people to Sabah, namely:

1. The period of British colonialism (British Empire) in Malay lands. The word Malay here covers the territory of eight kingdoms on the Peninsula, kingdoms in Sumatra and Kalimantan (Borneo, Sarawak, and Sabah regions). When Britain wanted to build infrastructure in Borneo, entrepreneurs needed workers from outside, including the Sabah region (Miyazaki, 2000). So, they took workers en masse from colonial territories in Vietnam and Indonesia.
2. During Dutch colonial rule, they collaborated with British private companies. The Dutch sent many ships to transport thousands of Javanese people to work in the fields, rubber plantations, and the manual labor sector in factories, manual laborers making roads, clearing land and ditches, etc. (Liow, 2015).
3. Japanese colonialism from 1942 to 1945, they also mobilized workers from Java for forced labor in the Japanese-occupied territories. The transfer was mostly in a rough and cruel way at that time. Many residents from Java were kidnapped and forced to move to become manual laborers in the Japanese-occupied territory of Borneo (Kamil and Noriah, 2011).

1880–1940 era, the wave of Javanese migration to Sabah continued under the orders of the North Borneo Chartered Company (Yahya et al., 2017). Until the mid-1930s, more than 30% of the workforce in Sabah came from Java, apart from workers from Sumatra, Sulawesi (Bugis-Makassar, Kalimantan, Madura, Banjar, etc.). The reason for employing people from Java was because they were seen as cheaper, easier to set up, and orderly, and they were also encouraged because of the law on the protection of natives (*Anak Negri Melayu*) and *Boemi Poetra*. After Sabah joined Malaysia (1963), Malay people of *Dhusun* descent, Bajau, and *Boemi Poetra non-anak Negeri* were socially divided into two, namely *Bumi Putra Anak Negeri* and *non-anak Negeri*.

The arrival of Javanese people in Sabah, especially in the Silama and Lahat Batu areas, was the result of cooperation between British Sabah companies and the Dutch Colonials. In 1880 it was recorded that there were 90 people (mostly female workers), from the port of Semarang to Singapore and then to Sabah. The development of the economic sector is the most visible cause of the influx of foreign workers into the Sabah region. Not all native people welcome the arrival of foreign workers and residents. They also stated that this was the cause of the damage to Sabah's economy. Jeffrey Kitingan, Sabah political leader stated:

*The presence of immigrants in the state has reached a critical level....unless the government is serious about solving the problem, the process of a political takeover could occur soon. This is like the political Trojan Horse preparing for the reserve takeover (Malaysiakini*, *2006* *in Dollah et al.*, *2024**)*.

#### 1.1.2 Characteristics of Javanese names

The name Javanese is currently used by millions of people and has spread to all corners of the world. Widodo (2010) states that although Javanese names have interesting characteristics, it is inevitable that Javanese names also develop over time. Determining certain limits for drawing on Javanese naming laws is difficult because, in reality, Javanese names also include many external factors and elements. However, according to research reports by Suranto (1983), Suharno (1987), Daljuni (1997), and Widodo (2010), the characteristics of Javanese names can be stated as follows.

1. Javanese names are often marked with the ending a [C], and a [A] for male names and i [I] for female names, apart from the mixed ending I [I].
2. The presence of a central element (independent morpheme) in the form of a word or name with the cloud Su-/Sa-/Sri-/Wi-, and the suffix an for the name male and prefix Su- and suffix -i for female names (morphemes are not independent), see Table 1.
3. The number of syllables varies between two and five, with the number of name syllables ranging from 1 to 8 name elements.
4. Not recognizing clan or family names, some indicated hereditary names.
5. Simple and easy to remember, referring to beauty, nature, character, and natural elements.

**Table 1:** Some examples of non-independent morpheme components.

**Su-**	**Sa-**	**Sri-**	**Wi-**
*Suharto* “very rich”	*Sateja* “shines”	*Srigrak* “vibrant”	*Wiguna* “good use”
*Sukur* “thankful”	*Satiti* “meticulous”	*Heroine* “Harjuna”s wife”	*Wireja* “hustle”
*Suci* “holy”	*Saputra* “a child”	*Srinata* “Javanese song”	*Wiranto* “courageous”
*Surti* “careful”	*Sasalancana* “moon”	*Screening* “essence”	*Winingsih* “good woman”

Source: Widodo (2010).

#### 1.1.3 The meaning of Javanese proper names

The name of the Javanese is actually one of the many living pieces of Javanese literature, an object of interpretation that continually changes and shifts according to the sign it refers to, based on space and time. It is not easy to draw a conclusion or theory about Javanese names because it requires a long period of time and caution to be able to explain the meaning of names adequately and perfectly. The meaning of names tends to be open (inconclusive) in concepts that tend to be stable. Javanese names are linguistic constructions that continually shape and reshape the Javanese 'world' in ways that are open and interesting for further study.

In reality, we always encounter different name themes. This matter can be revealed by knowing the thematic meaning, namely the meaning that appears as a combination of the meanings of the parts. The meaning of a name is communicated according to how the name giver organizes the message (Leech, 1983). An explanation of Leech's theory can be seen in Table 2.

**Table 2:** Seven types of meaning according to leech.

**Meaning**				**Information**
1. Conceptual meaning or understanding			Logical, cognitive, and denotative content
Meaning associative		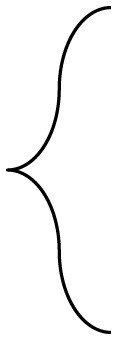	2. Connotative meaning	Communicated by what is referred to as language
				3. Stylistic meaning	Communicated from social circumstances regarding language use
	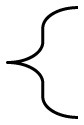			4. Affective meaning	Revealed by the feelings or behavior of the speaker/writer
			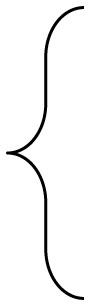	5. Reflective meaning	Conveyed through association with a different meaning than the same expression
					6. Collocative meaning	Conveyed through association with words that tend to occur in the range of other words
7. Thematic meaning					Communicated by arranging messages based on sequence and emphasis.

Source: Leech (1983).

## 2 Materials and methods

**Figure 1:**
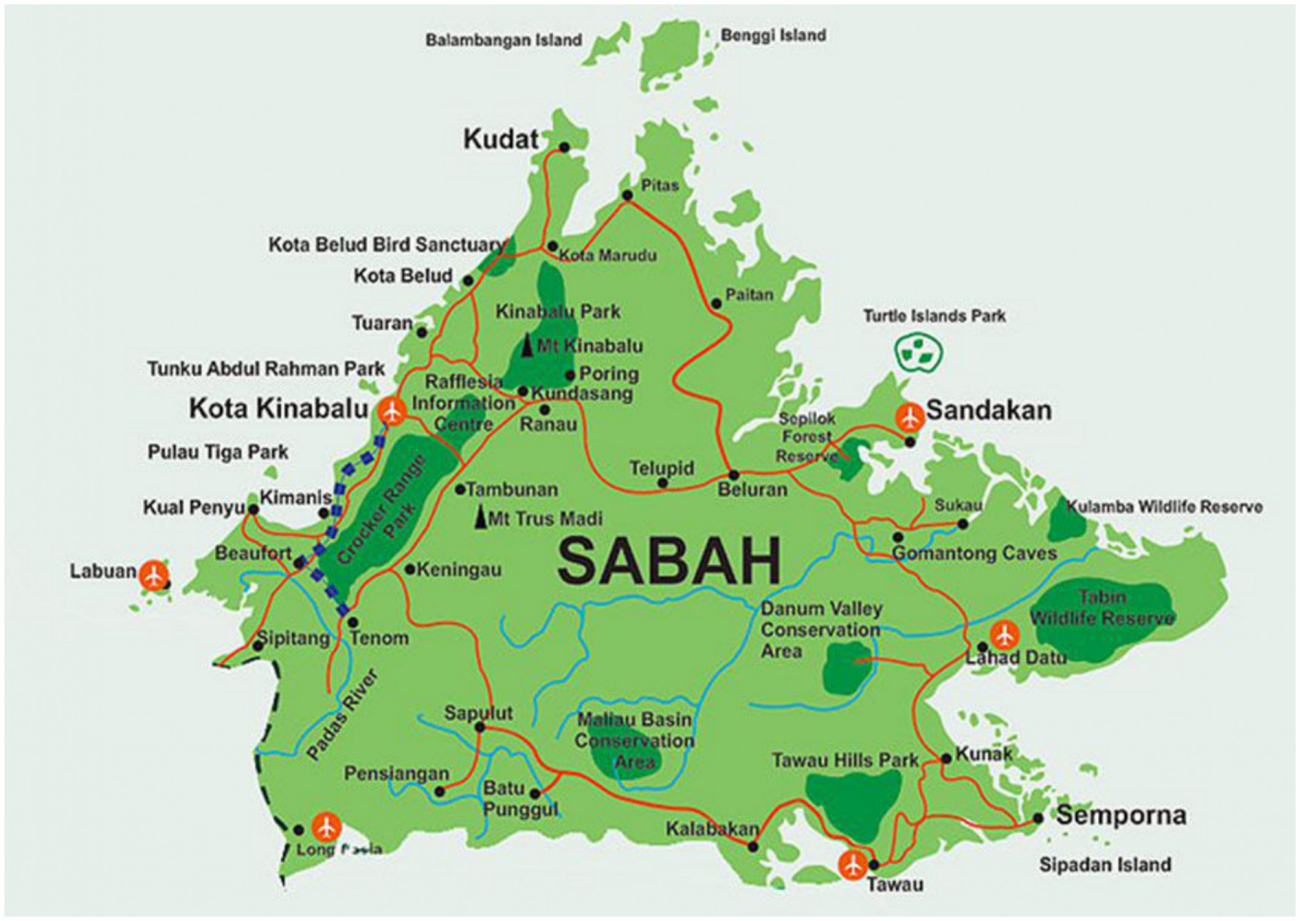
Map of Sabah city area.

This research involves registering the names of people in the Sabah region, especially the Kinabalu, Kundasang, and Keningau areas (For reference, see the map of Sabah in Figure 1). The reasons for conducting the research in these three locations are as follows: (1) The Sabah city area is predominantly inhabited by residents of Javanese descent (Razak, 2014), (2) The Javanese-descended community in Sabah is spread across these three cities and has attained certain social positions, particularly in the field of Islamic education, and (3) These conditions facilitate easier access to identifying key informants. Nevertheless, these three areas are also home to residents of Bugis, Banjar (South Kalimantan), and Madurese descent (in smaller numbers) (Daldjoeni, 1997). This reality, in fact, simplifies the process of data, source, and location triangulation, especially concerning various extra-lingual aspects, including historical, economic, security, political, and labor backgrounds (Dollah et al., 2024). The language used by people of Javanese descent in Sabah is generally Malaysian. However, in informal settings, the second and third generations (grandparents) still use Javanese as the language of conversation. Most children and teenagers already speak Malaysian.

### 2.1 Materials

The research data is a list of names collected from schools at various levels (302 names) and a list of names from the civil registration office in the Sabah region (404 names). The two sources are combined (706 names), and the data classification can be seen in Table 3.

**Table 3:** Classification and distribution of personal name data in research locations.

**Age**	**Kinabalu**	**Kudasang**	**Keningau**	**Σ**	**%**
3–10	64	14	10	88	12.5
11–20 years old	66	24	32	122	17.3
21–35	98	40	58	196	27.8
36–60	151	41	30	222	31.4
>60 years old	44	20	14	78	11
				706	100

The characteristic of the information obtained from the register of proper names is knowledge regarding the linguistic construction of names of the Javanese diaspora in the Sabah-Malaysia region, which includes specific ethnic patterns, diversity of elements, meanings of proper names, and characteristics of up-to-date proper names. This is the reason why a register of proper names is needed that includes the names of grandfathers, grandmothers, fathers and mothers to determine the onomastic landscape over several generations.

### 2.2 Method

The list of names obtained apparently continues to grow. During interviews with several informants, several other names were revealed, which could become important data in the research. Interviews were conducted seriously and in detail using structured (formal situations) and unstructured (informal situations) models for individuals and groups using the Focus Group Discussion (FGD) model. Each FGD was conducted 3 times, and each was attended by 6–8 people to find out the social, historical, and cultural background related to the development of the form and meaning of proper names used by the Javanese diaspora community in Sabah. Of the 23 informants, there are 5 key informants who have social positions in Sabah society from religious groups, academics, cultural figures, historians and ordinary people. Interviews with key informants were conducted to dig deeper into name motivations, expectations, and cultural tastes. Analysis in research is divided into two: social analysis and linguistic analysis. In fact, the two are closely related because the birth of new names cannot be separated from social and cultural developments and people's tastes, which move very dynamically in life.

## 3 Results

### 3.1 Development of the DJS personal name

The development of the name DJS can be traced from various angles, namely from history, socio-economic development, state politics and culture in a broad sense. The dynamics of the development of the name KJS are related to two issues, first, the process of social interaction of Javanese people with British and Dutch entrepreneurs (before and after the joining of Sabah to Malaysia), and the interaction of Javanese immigrants with local residents in the Sabah region (Kinabalu, Kudasang, and Keningau). In Table 4, it can be seen that the key informants of this research turned out to be very hetero-social. There are people of Javanese descent living together with immigrants from Bugis-Sulawesi, Riau, Palembang, Aceh, Padang, and a small community from Madura. This process of social interaction greatly influenced the emergence of changes in the form of Javanese names into Malay Islamic (Malaysian) names. Second, social interdependence namely the process of mutual benefit and influence between group members. The process of interaction and interdependence leads to efforts at adjustment, mutual influence, and understanding in conditions that are constantly moving, developing, and adapting to circumstances.

**Table 4:** Characteristics of key informants in research.

**No**	**Name**	**Gender**	**Age**	**Social position**	**Residence**
1	*Eko Suprayitno*	Male	43	USM academic	Kudasang
2	*Datuk Sobri*	Male	55	UiTM Academics	Kinabalu
3	*Mufti Ali Rosli*	Male	72	Perjasa Sabah	Kinabalu
4	*Noorasyimah*	Female	47	Public figure	Keningau
5	*Ruslindah*	Female	48	Associate Professor	Kinabalu
6	*Usop Bin Ali*	Male	55	Social Employee	Kudasang

Starting from these conditions, this research needs to consider several things related to the social phenomena behind the arrival of Javanese people in Malaysia, the social strategies that emerged in the adaptation process, and modern-global influences (name trends) to date. The three of them are considered to be able to provide an adequate explanation regarding the development of DJM's name to date.

### 3.2 The phenomenon of the arrival of the Javanese in Malaysia

The wave of Javanese people coming from various regions to the Sabah region actually occurred centuries ago (see Table 5). The various ethnic groups who came to Sabah (including Sarawak and Kuching) had different aims and objectives (Magiman et al., 2020). This difference in the purpose of arrival is apparently not the same as the movement of foreign people to the Peninsula (Malaysia). Especially what happened in Sabah, the reason for the collective arrival of the Javanese tribe was due to:

1. Reasons for working, migrating, looking for a better life. Javanese people from the districts of Semarang, Wonogiri, Yogyakarta, Klaten, Cirebon, Magelang, and Slawi flocked to the worker registration office near Semarang Harbor. In many cases, they are also “forced” to participate by other parties.
2. The reasons for trading were various forms of commerce; they socialized and interacted with Indigenous people, lived and eventually settled, and received recognition from the authorities (British Empire and local, regional administrators) together with trading groups from other ethnic groups.
3. The spread of Islam, this community came from religious groups (Islamic boarding schools). There are many *muftis* who give *fatwas, da'i* (preachers), and *ustad* (teachers). came to Sabah to spread Islam. They built mosques and *surau* and established Islamic learning centers in Sabah.
4. In the past, people were also found who had fled or sought refuge from the pursuit and threats of the Dutch and British colonial governments in their areas of origin in Java.

**Table 5:** Labor immigration from Java 1914–1918 in Sabah.

**Year**	**Male**	**Female**	**Children**	**Total**
1914	1	21	31	52
1915	3	14	33	50
1916	3	20	10	33
1917	1	94	85	180
1918	1	59	73	133
Total	9	208	232	449

Source: Orang Jawa di Borneo Utara, Satu Nota Awal (Tamrin, 1988).

The arrival of Javanese people in Sabah, especially in the Silama and Lahat Batu areas, was the result of cooperation between British Sabah companies and the Dutch Colonials. In 1880 it was recorded that there were 90 people (mostly female workers), from the port of Semarang to Singapore and then to Sabah. Since then, >2,000 people have been sent to Sabah every year, except for those who died or got sick on the way, so they did not reach their destination.

**Figure 2:**
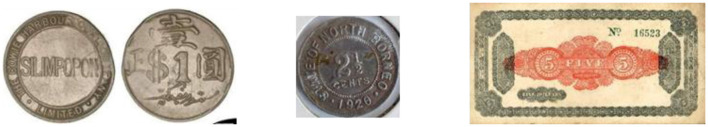
Shari's personal collection, Tokens, special currency coins for Javanese field workers in Sabah.

There is an intriguing phenomenon: initially, the Javanese workers used a contract system and with a special currency (see Figure 2), so many managed to return to Java on Dutch Royal ships such as the Royal-Inter Ocean. In 1907, there were 8,449 workers, and 5,068 managed to return home to Java. This means that there are some who continue to extend their contracts, or stay in Sabah, enter a *dhusun*, get married, and start families with state children or non-state children. A small number married Sabah natives.

Since 1920, sending Javanese workers to Sabah has decreased due to the world economic crisis. And it became a phenomenon again in WWII, but by the Japanese invaders. Then there was a wave of Islamic religious teachers arriving because of Javanese Sabah contacts with *ustads* and preachers from Java. After the joining of Borneo with the Royal Malaysian Government on the Peninsula, the wave of teachers/*ustad*/*da'i* became bigger, due to the existence of the Malaysian Kingdom Politics (PM. Tungku Abdul Rahman). In the 1940s, 1950s, 1960s, and early 1970s, many teachers came from Java and settled in Sabah.

“*The issue of immigrants in the country of Sabah is a complex issue and involves many parties so that it gets a complex response too... it is not only related to economic issues but is related to the conditions prevailing in Malaysia” (Dollah et al.*, *2024**, p. 80)*.

“*There are many immigrants from Java, especially Cik Gu (clerics, teachers) and Da'i, who are active in spreading and developing Islam in Sabah. Compared to 1970–1980, the number of Muslims on Saba has increased by more than 70%. This indicates that there are different reasons apart from economic reasons for them coming and staying in Sabah (Datuk Sobri/interview/12 June 2024)*.

The arrival of people of Javanese descent falls into three categories, namely (1) entry with complete documents and permit documents, (2) entry without complete permit requirements, and (3) escape, worker smuggling. Specific data shows that there have been no deportations of Javanese workers since the 1990s. However, they are included in the category of immigrants from Indonesia. As shown in Table 6.

**Table 6:** Deportation of migrants from Indonesia to Sabah.

**Year**	**Indonesia**	**Others**	**Return voluntarily**	**Amount**
1990–2011	161,097	4,016	74,210	90.903
2012–2013	5,962	433		6,395
2014	3,624	345		5,969
2015	6,055	220		6,275
2016	3,955	231		4,186
Amount	180,693	5,245	74,210	93,267

Based on the data above, for the Javanese diaspora in the Sabah region, it is very important to have the spirit and ability to adapt socially and culturally. The encounter with the relatively different traditions, culture, and life behavior of the Bumi Putra and Sabah Malaysians, whose tranquility turned out to be the main concern of the Javanese diaspora. They make every effort to be accepted and live together peacefully. Obtaining recognition from the Sabah government, obtaining permission to stay, and obtaining full rights as a non-indigenous son of the earth, were truly the desires of the Javanese diaspora at that time.

*Since they first set foot in Sabah (1940s), their grandparents from Java were aware of themselves as immigrants, earning a living and deciding to live in Sabah. So, there is no reason why we should not follow the rules, procedures, traditions, and culture in Sabah. Now, we feel like Malaysians of Javanese descent. We are loyal to the rules and laws of Sabah, even though biologically, we are of Javanese descent. However, our children marry Sabahans (Roslindah/interview/11 May 2024)*.

### 3.3 Proper names and DJS adaptive behavior

One of the most easily recognized DJS identities is from its name. Until the 1950s, DJS people still used Javanese names (Suharto, 1987). Except, some forms of names have been changed by regional registrars, worker archive administrators, registrants of prospective contract workers, etc. However, its original form can still be traced. From documents available at the Civil Registration Office in the Kota Kinabalu and Kudasang areas (1988), several DJS names were found that may have been changed, including:

Table 7 shows that changing DJS names to Malay Islamic names is a model of socio-cultural adaptation. These changes occur evolutionarily in line with social and political developments, community history, position, and citizenship processes. The long process of adaptation and mutual acceptance between the local population and the migrant collective occurred well and “melted” in the form of a proper name.

“*There are many reasons why we chose Malay Islamic names for my children. First, cultural considerations. Children will grow and develop with a local culture, so there should be no cultural obstacles for them to face. Second, considerations in mixed marriages are because my wife is from Malacca, and she wants her children to have Malay names so that they can be accepted by their relatives in Semenanjing and Sabah. Third, there is influence from teachers, ustad, and preachers from the Peninsula, especially here. Many come from Malaya, from Ranau. Obviously, they want their generation to use Islamic names. And, apparently, this later became a model as a form of adaptation of DJS in Sabah*.” (Mulio Gariman/interview/16 June 2024)”.

**Table 7:** Changes in the form of the DJS name.

**The original name**	**Change of name form**	**Year of arrival**	**Place of origin**
*Partinah*	*Fatimmah*	1933	Sampung, Ponorogo, Central Java
*Sabar*	*Shaobari*	1942	Gumelar, Banyumas, Central Java
*Kariman*	*Gariman*	1937	Ajibarang, Central Java
*Kliwon*	*Kaliwoon*	1948	Sukorejo, Wonogiri
*(Su-)Tarjo*	*Tarjoh*	1933	Wedi, Klaten, Central Java
*Trimo(a)*	*Tarimoh*	1943	(?), Sragen

The results of the interview were developed by examining existing documents so that the factors that influenced the name change could be identified, including factors from the Royal Malaysian Government and certain social movements. Several social phenomena that have been recorded show the existence of four other social factors, namely:

1. In the early days, in 1963, after the merger of Sabah into Malaysia, there was no official law regarding religion in the state of Sabah. The immigrants (including DJS) still maintain their own cultural traditions, including the use of original Javanese names.
2. In 1971, the Islamic religion was included in the constitution, and the official religion in Sabah is Islam. This political movement and new legislation gave birth to a new socio-cultural evolution. The DJS community began to adapt socially and culturally. In fact, this official regulation further accelerated the transfer of the status of Javanese residents to Javanese Malays.
3. The DJS community is included in the Malay group along with other collectives such as Sulawesi, Banjar, Aceh, Padang, etc. This phenomenon was a strong driver for the name change.
4. The influence of the Islamization movement on the Peninsula: since the early 1970s, Javanese names have been changed or deliberately changed by civil registration officers in order to facilitate the process of social adjustment, land ownership, and population status in Sabah. Most name changes occur in schools.

### 3.4 Construction of the personal name of the Sabah Javanese diaspora (DJS)

The name DJS is an utterance that has an interesting form, structure, and meaning (Widodo et al., 2023). Characteristics that stand out in terms of name writing include (1) the Linguistic Construction of NDJM, (2) the Syllabic Patterns of the name DJM, (3) the Diversity of elements of the name DJM, and (4) the Meaning of the Name DJM. The four characteristics are connected to each other even though they are each. The scope of the study can be differentiated, but they are interrelated to form a unique form, meaning, and significance, as the name DJM.

### 3.5 DJS linguistic construction

When researching the DJS name data list in 1950–1960, we found many forms of the names *Nur* “light”, *Siti* “woman/mother”, and *Zahra* “blooming”, “flower of the world”, which were popular as elements of women's names. The elements of the names *Muhammad, Abdul*, and *Afiq* are popular as elements of male names in their complete form. This name element in Table 8 is found in the names of children and adults, which have a basic construction in the form of a single or monomorphemic noun (Widodo et al., 2023; Uhlenbeck, 2008). In more complex forms, combined variants are found, namely monomorphemic patterns combined with other basic morphemes to form new polymorphemic name elements.

**Table 8:** Process of changing Monomorphemic to Polymorphemic constructions.

**Monomorphemic elements**	**New forms of polymorphemia**	**Information**
*Awang, Ali*	*Awangalian*	Addition of the ending *-an*
*Nur, Haikal*	*Nurhaikal*	Complete merger
*Fikri, Allah*	*Fikrirullah*	The vowel/a/becomes/ru/
*Janah, tun, naim*	*Janatunnaim*	Missing consonant/h/

Monomorphemic construction of community names in Sabah is unique because it appears in almost all DJS names today. This means that name data taken from 1950 to 2020 still shows the same tendency, namely the combination of several forms of monomorphemic names. For example, the name series *Siti Naaila Qisya Binti Ahmad Kurnawan* (8), *Nur Aisyah Binti Siman* (12), *Mod. Haidar Bin Yahya* (39)*, Usop Bin Ali* (54), and *Dollah Sabari Bin Wongso* (66) show the same structure. Although there are exceptions to the name elements *Sabari*→*Sabar* + *i* and *Kurniawan*→*Kurnia* + *wan*.

Monomorphemic and polymorphemic name forms show that there has been a change and development in the form of the DJS name from the original Javanese name construction, which apparently had occurred over the previous seven decades. The same reality occurs in Peninsular Malaysia, namely *Johor, Melaka*, and *Selangor* (Widodo et al., 2023). In the 1950s/1960s, the name DJS of Malaysians of Javanese descent still showed a typical Javanese construction, namely a combination of the independent morpheme and the non-independent morpheme (*Su*-), as shown in Table 9.

**Table 9:** Polymorphemic construction of DJS name old age group.

**Name (age)**	**Morphemic processes**	**Meaning of name**
*Sudirman Bin Danto* (72 years)	*Su + dirman;* “good, deed”	A man who has good actions and attitudes
*Sukanti Binti Mana* (78 years)	*Su + karti*; “Good (living) water”	A woman with a good face and appearance
*Suratmo Bin Bai* (71 years)	*Su +(r)atmo*; “Good boy”	A man of good birth.
*Suwignyo Bin Midar* (69 years old)	*Su + Wignyo*; “clever and kind”	Have kindness and good behavior.

**Scheme 1:**
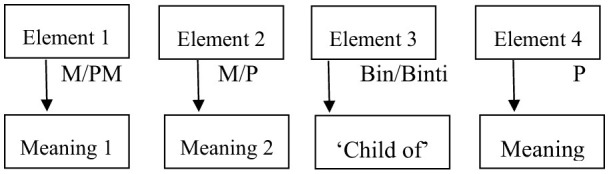
Construction of the Bantuk form and the meaning of the DJS name.

Based on name data in the field (see Table 9), the DJS community prefers to use monomorphemic name constructions compared to polymorphemic ones. This construction influences the pattern of meaning that is built (Mudrikah, 2015; Uhlenbeck, 1982). In this way, the meaning can be drawn: a name consists of one or more elements that make it up. The meaning of a polymorphemic (complex) name element is built by the meaning of its elements, either monomorphemic or polymorphemic, which can be explained in Scheme 1.

For the record, the construction of the form and meaning of the proper name DJS has developed over time. This development includes form (phonetics) and meaning (semantics). The forms of old names given and inherited by Javanese ancestors have changed their construction to become names typical of the country of Sabah, which is also different from Malay names in Peninsular Malaysia, of course, through methods that are also classified as special.

### 3.6 The syllabic pattern of the DJS personal name

The syllabic pattern of DJS's name is one of the interesting problems in this research. The real aim is to (1) ensure that the length and shortness of the name describes a certain pattern that connects the parts, (2) find out the beauty of the name DJS linguistically, and (3) critically reveal the model of meaning, and interpretation approach of a name by paying attention to the form units in speech (Suharno, 1987; Verhaar, 1998). From the data collected, it is clear that the name DJS is quite varied. From 706 names from various ages, we get the impression that modern names given to those born after the 1970s are more interesting because there are so many variations, and each one shows a certain meaning, which is a pillar of the spirit of the times.

Table 10 shows that DJS name elements mostly consist of one to four syllables which tend to be found in modern names (after 2010). This finding is impressive because it is somewhat different from the names of people of Javanese descent in the Peninsula region, but it also shows a different direction to the development of Javanese names in Indonesia. Modern names in Java tend to be long, there are even many names consisting of five and six syllables (*Sulistyaningrum, Widyariningtyas, Damaringalam*, etc.). This difference is quite natural when viewed from the historical, social, and cultural backgrounds. Isn't a name formed by its context?

**Table 10:** Number of syllables for DJS name elements.

**Syllables**	**Male names**	**Female names**
One	*Mohd. Muh., Nur*	*Nur, Noor, Sir*,
Two	*Omar, Zaky, Rayyan, Azlan, Azrin, Afiz, Afiq*	*Azra, Uzma, Hanna, Alya*
Three	*Ghazali, Tony, Ummar, Hamam, Fatahul*	*Aqilah, Naailla, Ezuma, Hamidah, Hidayati*
Four	*Hafizuddin, Zahrulnizam, Norashidi, Hishamuddin*	*Qaisahrah, Qairina, Umairah, Nurfareena*

### 3.7 The diversity of the elements of the DJS personal name

The name of the people of Javanese descent in Sabah (DJS) has many elements taken from very diverse sources. This source is very important to know as a label, sign, and identity of a person or group that differentiates it from other people (Hofmann, 1993; Lehrer, 1999). In contrast to Javanese names in the Peninsula region, the name DJS does not contain many elements of the name Muhammad, which is single-oriented toward the Prophet Muhammad as a male name. However, the form of use still exists, but in abbreviated form (acronym) to *Mohd, Moch*, and *Muh*. Additionally, the elements of the names *Noor, Nur*, and *Siti* are for women's names (Widodo et al., 2023).

This is clear evidence that names are a subject of language that is greatly influenced by the context, situation, conditions, and circumstances of their use. The Malay community in Peninsular Malaysia has long embraced religion strongly. This situation is different from the country of Sabah. In fact, Islam became the majority religion only after the 1970s. This knowledge can be obtained from the names that people bear in each decade of the era. The diversity of the elements of the DJS name cannot be separated from the corridors of norms, traditional conventions, bonds of national spirit, the power of adaptation, the spirit that is built, and the cultural tastes of the people (see Tanz, 1982).

It turns out that this proves the theory. This is easy to understand because Malaysia is an Islamic country, and the majority of the population is Muslim. However, it is interesting that the writing of the elements of the name *Muhammad* has several acronym variants, namely: *Muhammad* (form still intact), *Muhamad* (written with one “m”), *Moch., Mohd*, and *Mohamad*. Likewise, what happens is that the element of the name *Nur* 'light' appears in several variants, namely *Nor* and *Noor*. Interestingly, the element of the name ‘*Nor*' often appears as a component of the names *Noriah, Normah, Norwadiah, Norakmar*, and *Noraini*. This also often happens in name series in Java, Asia, and Europe because proper names are a social product (Frawley, 1992).

Table 11 is a classification of DJS name elements based on data on the names of the Malaysian Javanese diaspora community in Johor, Melaka, and Selangor by grouping them based on similarities in form, nature, and meaning, which shows the same syntactic behavior and nature of relationships (cf. Alford, 1988). Based on the data collected, the elements of the DJM name can be classified into seven categories as follows.

**Table 11:** Classification of 7 DJM name categories.

**Name source**	**Original form**	**Transformation**
*Asma'ul-Husna*	*Arrahman* “gracious”*, Arrahim* “caring”*, Almaliku* “to rule”*, Alghafuru* “forgiving”,	*Abdul Rochman Bin Sobri Abdol Rokim Bin Satiman Malik Husni Bin Wagya Moch. Abdul Goffur*
Names of the Prophets	*Adam, Idris, Ibrahim, Ismail, Ishaq*	*Adam Wahida, Idris Sardi Bin Jamek, Ismel Hassan Bin Toir, Modh. Iskak Bin Rosmin*
Companions of the Prophet Muhammad	*Abu Bakar Ash-Shiddiq, Umar bin Khathab, Utsman bin Affan, Ali bin Abu Thalib*	*Sidiq Airrur Jhan Bin Dirman, Usman Arief Bin Hisham, Yussuf Alie Bin Darmin, Ali Masqurri Bin Djaki*
Ten people are guaranteed to enter Allah SWT's heaven	*Sa'd bin Zaid bin Naufal, Abu Ubaidah Amir bin Al-Jarrah, Sa'd bin Abi Waqqash, Abdurrahman bin Auf, Zubair bin Awwam, Thalhah bin Ubaidillah*	*Bachtiar Said Bin Wongso, Much. Ubaidillah Bin Sapon, Saad Bin Darwis, Ammar Abdol Rahman Bin Ali, Dollah ZubairBin Kirman*,
The Prophet's Wives	*Khadijah, Saudah, Aisyah, Hafshah, Zainab, Ummu Salamah, Juwairiyah, Shafiyah*	*Siti Qodijah Binti Misran, Aina Saudah Binti Yussri, Nurul Hasyah Binti Nur, Auliya Zenab Binti Yahkub, Salammah Zuhri Binti Atin, Zara Safiyyah Binti Khuzni*
Sons and daughters of the Prophet	*Al-Qasim, Abdullah, Ibrahim, Zainab, uqayyah, Ummu Kultsum*, dan *Fathimah*	*Kazim Dayani Bin Ya'akob, Ibrahim Hatta Bin Jumaat, Rukayyah Binti Osman, Dynna Binti Ismadi, Fatimmah Assaqui Binti Yajid*

## 4 Discussion

The basis used to reveal the deepest meaning of the name DJS is related to the frame of reference used based on language and culture, codes and conventions, and special matters (Gardiner, 1954; Ullman, 1977; Crystal, 1987). The frame of reference is structured in such a way that it refers to (1) identification, (2) uniqueness, (3) denotation and connotation, (4) distinctive sounds, and (5) grammatical criteria (Widodo et al., 2023). From the existing DJS name data, the five reference elements are summarized to explain the problem of the meaning of the DJS name as follows.

It is increasingly clear that the name DJS is related to the history of its owner, important recorded phenomena, developing knowledge, truth, meaning, and the special ways the people of Javanese descent in Sabah overcome their problems. These empirical facts clearly indicate that the changes in form and meaning of DJS personal names represent an adaptive strategy for shaping the self-identity of postcolonial communities, as well as the social and cultural responses of DJS to various migration policies that have led to cultural hybridity in the Sabah region.

There is a strong connection between the meaning of the name DJS and prayers, hopes, and desires, which become a “myth of liberation” from psycho-social pressure like a migrant. The occurrence of changes in the form of names and changes in the meaning they contain are truth conditions that are considered the most appropriate (Langendonck, 1985).

Meaning, as an important part of language, proper names have their own uniqueness. Related to this Ullman (1977) and Widodo et al. (2023) state that the person's name identifies and does not signify 'understand.' So, the stronger understanding of Islam in the country of Sabah can be measured by the names used by society today. This is the main way that occurs in the name DJS; it can be ascertained that the majority of DJS adhere to the Islamic religion. This cannot only be proven from the linguistic aspect of the name. But also, the historical, social, and cultural context.

The meaning of the name DJS is understood from its denotation and connotation. The name *Siti Naaila Qisya* is very difficult to mark as a name with Javanese influence. However, if accompanied by his father's name, the influence is clear. *Siti Naaila Qisya Binti Ahmad Kurniawan*. The endings -wan, -no, -to, and -di clearly refer to the Javanese form of the name. As it becomes clearer, it turns out that the meaning of a name does not refer to what it denotes, but to what it connotes.

The basic meaning of a name can be called a 'distinctive' meaning because, in principle, one name is for one person only. There are many elements in names that are the same and, of course, have the same meaning, but it turns out they have different meanings for their owners. Proper names have grammatical specificity because even though they are included in the study of linguistic grammar, they are actually ungrammatical, as well as the components of names and their elements.

Based on the results of the analysis of linguistic aspects, it can be seen that the basis for choosing the elements of the name DJS, namely (1) religion and belief, (2) social and cultural considerations, (3) considerations of lineage, origin, family, or historical setting of the bearer, and (4) accommodate all the hopes, desires and requests of parents.

Proper names are a phenomenal subject of linguistic research. Why? Several studies show that there is a strong relationship between linguistic phenomena and other factors, namely social, historical, and cultural. DJS's proper name shows strong evidence. Similar to the personal names of people of Javanese descent in the Peninsula, the name DJS has clear characteristics, including: (1) has a clear gender marker with the addition of the name *bin* for male names, and *binti* for female names behind the person's personal name. The series of DJS names rarely include personal names with mixed gender markers. In Java, the name element “*Agus*” (derived from the month of August) is a mixed-gender marker. The name “*Agus*” can be used for females with various modifications (*Agustina, Agustia, Agustine, Agustin*). This form of name is rarely found in Peninsular Malaysia or in the Sabah region. (2) DJS's self-name shows the image of Java and Islam. This is proof of the success of the DJS Community's adaptation efforts in the places they live. (3) Since the 1970s-1980s, Javanese names have shifted to typical Malay Islamic names. On the other hand, the forms of Javanese names are also written differently with different pronunciations (*Airiz, Darwisy, (A)Iszmy, Yussrie*). In comparison, in the Peninsula region, the use of the consonant/z/is relatively dominant, especially in modern names (*Zahid-Sahid, Syazuwin-Aswin, Syazwana-Sas(r)wana*, and *Izdihar-Ishar*).

The name DJS grew and developed in line with the history of Sabah. From the description that has been presented, it turns out that the name DJS is closely related to motivation and encouragement, thought patterns, and cultural responses with historical, social, and cultural backgrounds. It is increasingly clear that changing the name of DJS is a “subtle way” to carry out humanitarian diplomacy, which has succeeded in erasing the “black page” of the history of the arrival of Javanese people in Sabah for centuries. Currently, the DJS collective has succeeded in having a relatively much better level of prosperity. It's natural for some DJS people to state the reason why they changed their name? There is an effort to give a better name, namely a name that is adaptive to environmental conditions in accordance with the phenomena of the times. So, the name association shifts from the meaning of safety to (a) Smart, knowing correctly the ways and efforts to live a healthy, prosperous, and sufficient life; (b) Rich, having a clear economic and social position (c) Pious and wise so that you can live an orderly life, (d) Graceful and has 'competitiveness' with other names, (e) dignified.

Nonetheless, this study has examined DJS names in terms of form, meaning, significance, and the intention behind their naming. Social and cultural influences are clearly evident in DJS names and are undoubtedly part of a cultural adaptation strategy. Further research is recommended to carefully and critically explore the psychological impacts of DJS name changes on name bearers and the wider community, particularly in the fourth or fifth generations. This is important as input for policymaking and onomastic studies in general.

## Data Availability

The original contributions presented in the study are included in the article/supplementary material, further inquiries can be directed to the corresponding author.
